# Supplementary material for: High-Throughput Microfluidic Real-Time PCR for the Detection of Multiple Microorganisms in Ixodid Cattle Ticks in Northeast Algeria
Source: Pathogens. 2021 Mar 18;10(3):362. doi: 10.3390/pathogens10030362 (PMC8002991; doi:10.3390/pathogens10030362)
Supplement: Supplementary file 1 [file pathogens-10-00362-s001.pdf]

**Table S1**. Detection of single and co-infections with microorganisms in cattle ticks.

| Microorganism species | | N° | Tick species | | | | | | | | |
|---|---|---|---|---|---|---|---|---|---|---|---|
| | | | *Rhipicephalus* spp. | *R. bursa* | *R. sanguineus* | *R. annulatus* | *Hyalomma* spp. | *H. detritum* | *H. marginatum* | *H. lusitanicum* | *I. ricinus* |
| **Single infection** | *Borrelia* sp. | 2 | 1 | 1 | 0 | 0 | 0 | 0 | 0 | 0 | 0 |
| | *A. marginale* | 1 | 1 | 0 | 0 | 0 | 0 | 0 | 0 | 0 | 0 |
| | *R. aeschlimannii* | 6 | 1 | 5 | 0 | 0 | 0 | 0 | 0 | 0 | 0 |
| | *R. massiliae* | 1 | 1 | 0 | 0 | 0 | 0 | 0 | 0 | 0 | 0 |
| | *Rickettsia* spp. | 22 | 6 | 5 | 3 | 0 | 4 | 3 | 0 | 0 | 1 |
| | *Bartonella* spp. | 3 | 3 | 0 | 0 | 0 | 0 | 0 | 0 | 0 | 0 |
| | *B. bigemina* | 1 | 0 | 0 | 0 | 1 | 0 | 0 | 0 | 0 | 0 |
| | *T. orientalis* | 1 | 1 | 0 | 0 | 0 | 0 | 0 | 0 | 0 | 0 |
| | *FLE* | 10 | 4 | 3 | 0 | 0 | 1 | 1 | 1 | 0 | 0 |
| | **Subtotal** | 47/235 20% | 18 | 14 | 3 | 1 | 5 | 4 | 1 | 0 | 1 |
| **Dual infection** | *Borrelia* sp .+ *R. massiliae* | 1 | 0 | 0 | 1 | 0 | 0 | 0 | 0 | 0 | 0 |
| | *Borrelia* sp.+ *Rickettsia* sp. | 1 | 1 | 0 | 0 | 0 | 0 | 0 | 0 | 0 | 0 |
| | *Anaplasma* sp. + *Rickettsia* sp. | 1 | 1 | 0 | 0 | 0 | 0 | 0 | 0 | 0 | 0 |
| | *Anaplasma* sp.+ *R. massiliae* | 1 | 0 | 0 | 1 | 0 | 0 | 0 | 0 | 0 | 0 |
| | *R. aeschlimannii*+ *T. orientalis* | 2 | 1 | 1 | 0 | 0 | 0 | 0 | 0 | 0 | 0 |

| | | | | | | | | | | | |
|---|---|---|---|---|---|---|---|---|---|---|---|
| | *Rickettsia* sp. +*T. orientalis* | 2 | 1 | 0 | 0 | 0 | 0 | 1 | 0 | 0 | 0 |
| | *FLE* + *Rickettsia* spp. | 61 | 7 | 6 | 1 | 0 | 18 | 17 | 7 | 2 | 3 |
| | *FLE* + *R. monacensis* | 2 | 0 | 0 | 0 | 0 | 0 | 0 | 0 | 0 | 2 |
| | *FLE* +*R. aeschlimannii* | 8 | 1 | 1 | 0 | 0 | 3 | 1 | 1 | 1 | 0 |
| | *FLE* + *R. massiliae* | 5 | 0 | 0 | 0 | 0 | 3 | 2 | 0 | 0 | 0 |
| | *FLE* + *R. helvetica* | 1 | 0 | 0 | 0 | 0 | 0 | 0 | 0 | 0 | 1 |
| | *FLE* + *T. orientalis* | 1 | 0 | 0 | 0 | 0 | 0 | 1 | 0 | 0 | 0 |
| | **Subtotal** | 86/235 36.6% | 12 | 8 | 3 | 0 | 24 | 22 | 8 | 3 | 6 |
| **Triple infection** | *A centrale*+ *Rickettsia* sp. + *T. orientalis* | 1 | 0 | 1 | 0 | 0 | 0 | 0 | 0 | 0 | 0 |
| | *A. marginale* +*R. aeschlimannii, T. orientalis* | 2 | 1 | 1 | 0 | 0 | 0 | 0 | 0 | 0 | 0 |
| | *FLE* +*A marginale*+ *T. orientalis* | 1 | 1 | 0 | 0 | 0 | 0 | 0 | 0 | 0 | 0 |
| | *FLE* +*Borrelia* sp. +*R. massiliae* | 1 | 0 | 0 | 0 | 0 | 1 | 0 | 0 | 0 | 0 |
| | *FLE* +*Anaplasma* sp. + *T. orientalis* | 1 | 0 | 0 | 0 | 0 | 1 | 0 | 0 | 0 | 0 |
| | *FLE* + *Bartonella* sp. + *R. aeschlimannii* | 2 | 0 | 0 | 0 | 0 | 2 | 0 | 0 | 0 | 0 |
| | *FLE* + *Bartonella* spp. + *Rickettsia* spp. | 4 | 0 | 0 | 0 | 0 | 0 | 3 | 0 | 1 | 0 |
| | *FLE* + *Borrelia* spp. + *Rickettsia* spp. | 4 | 0 | 0 | 0 | 0 | 2 | 2 | 0 | 0 | 0 |

| | | | | | | | | | | | |
|---|---|---|---|---|---|---|---|---|---|---|---|
| | *FLE* + *Borrelia* sp.+ *R. aeschlimannii* | 2 | 0 | 1 | 0 | 0 | 1 | 0 | 0 | 0 | 0 |
| | *FLE* + *A. marginale* + *Rickettsia* spp. | 8 | 2 | 0 | 0 | 0 | 2 | 1 | 0 | 0 | 3 |
| | *FLE* + *Anaplasma* spp.+ *Rickettsia* spp. | 5 | 1 | 2 | 0 | 0 | 0 | 1 | 1 | 0 | 0 |
| | *FLE* + *R. aeschlimannii*+ *T. orientalis* | 2 | 0 | 0 | 0 | 0 | 2 | 0 | 0 | 0 | 0 |
| | *FLE* + *R. massiliae* +*T. orientalis* | 2 | 1 | 0 | 0 | 0 | 0 | 1 | 0 | 0 | 0 |
| | *FLE* + *Rickettsia* spp. +*T. orientalis* | 14 | 1 | 2 | 0 | 0 | 8 | 1 | 1 | 0 | 1 |
| | *FLE* + *R. massiliae*+ *B. bigemina* | 1 | 0 | 0 | 1 | 0 | 0 | 0 | 0 | 0 | 0 |
| | *FLE* + *R. helvetica* +*T. orientalis* | 1 | 0 | 0 | 0 | 0 | 0 | 0 | 0 | 0 | 1 |
| | **Subtotal** | 51 /235 21.7% | 7 | 7 | 1 | 0 | 19 | 9 | 2 | 1 | 5 |
| **Quadruple infection** | *A. marginale* + *R. aeschlimannii* + *T. orientalis* + *Bartonella* sp. | 1 | 1 | 0 | 0 | 0 | 0 | 0 | 0 | 0 | 0 |
| | *FLE* + *Borrelia* sp. + *A. marginale* + *Rickettsia* sp. | 1 | 0 | 0 | 0 | 0 | 1 | 0 | 0 | 0 | 0 |
| | *FLE* + *Borrelia* sp. + *T. orientalis* + *Rickettsia* sp. | 1 | 0 | 0 | 0 | 0 | 0 | 1 | 0 | 0 | 0 |

| | | | | | | | | | | | |
|---|---|---|---|---|---|---|---|---|---|---|---|
| | *FLE* +*Borrelia* sp. + *Bartonella* sp. + *R. aeschlimannii* | 1 | 0 | 0 | 0 | 0 | 1 | 0 | 0 | 0 | 0 |
| | *FLE* +*Borrelia* sp. + *T. orientalis* + *R. helvetica* | 1 | 0 | 0 | 0 | 0 | 0 | 0 | 0 | 0 | 1 |
| | **Subtotal** | 5/235<br>2.1% | 1 | 0 | 0 | 0 | 2 | 1 | 0 | 0 | 1 |
| | **Total** | 189/235<br>80.4% | 38/235<br>16.2% | 29/235<br>12.3% | 7/235<br>3% | 1/235<br>0.4% | 50/235<br>21.2% | 36/235<br>15.3% | 11/235<br>4.6% | 4/235<br>1.7% | 13/235<br>5.5% |
| | | | **75/109<br>68.8%** | | | | | **101/113<br>89.3%** | | | **13/13<br>100%** |

N°: number of ticks *FLE: Francisella*-like endosymbiont.
